# New species records of *Culicoides* biting midges (Diptera: Ceratopogonidae) for the state of Rondônia in Brazilian Amazon

**DOI:** 10.3897/BDJ.5.e13075

**Published:** 2017-05-09

**Authors:** Luis Paulo Costa Carvalho, Emanuelle de Sousa Farias, Luiz Herman Soares Gil, Felipe Arley Costa Pessoa, Jansen Fernandes Medeiros

**Affiliations:** 1 Programa de Pós-graduação em Biologia Experimental, Universidade Federal de Rondônia, Porto Velho, Brazil; 2 Laboratório de Entomologia, Fundação Oswaldo Cruz, Fiocruz Rondônia, Porto Velho, Brazil; 3 Laboratório de Ecologia de Doenças Transmissíveis na Amazônia, Instituto Leônidas e Maria Deane, Fiocruz Amazônia, Manaus, Brazil; 4 Instituto de Pesquisa de Patologias Tropicais, Porto Velho, Brazil

**Keywords:** Rondônia State, new records, Amazon.

## Abstract

**Background:**

*Culicoides* biting midges are small insects that are proven vectors of pathogens that cause disease in animals and humans. There are 1,368 species of *Culicoides* in the world, including 149 species in Brazil and 122 species in the Brazilian Amazon Basin. This study documents specimens that were collected between 2013 and 2015 in the municipalities of Alvorada d’Oeste, Buritis, Cacoal, Costa Marques, Espigão d’Oeste, Guajará-Mirim, Pimenta Bueno, Porto Velho and São Francisco Guaporé. Collections were performed using HP light traps in forest, pasture and peridomicilie environments.

**New information:**

Species newly recorded in Rondônia State include *Culicoides carpenteri* Wirth & Blanton, 1953; *C. dasyophrus* Macfie, 1940; *C. eublepharus* Macfie, 1948; *C. galindoi* Wirth & Blanton, 1953; *C. heliconiae* Fox & Hoffman, 1944; and *C. ignacioi* Forattini, 1957. This is the first record in Brazil of *C. darlingtonae* Wirth & Blanton, 1971.

## Introduction

The biting midges *Culicoides* Latreille, 1809 (Diptera, Ceratopogonidae) are small insects; their blood-feeding females bite birds and mammals, including humans, and they sometimes feed in large swarms. The bites are painful and can cause severe reactions. These insects can also transmit pathogens that cause disease in humans and domestic animals of economic importance (Ronderos et al. 2003, Borkent and Spinelli 2007). The diverse species of *Culicoides* are distributed around the world; currently, 1,368 species have been described worldwide; 149 species have been recorded in Brazil and 122 species have been recorded in the Brazilian Amazon Basin (Borkent 2016a, Carvalho et al. 2016, Farias et al. 2016a, Farias et al. 2016b, Santarém and Felippe-Bauer 2017). Despite evidence of high species diversity of *Culicoides*, only a few surveys have been conducted in the Brazilian Amazon Basin, primarily in the states of Amazonas and Pará. Recently, Carvalho et al. (2016) made collections of *Culicoides* species near Porto Velho, the capital of Rondônia State; they recorded 28 species, thereby increasing the number of species recorded in Rondônia to 33. Herein, we provide seven new records of *Culicoides* species collected in Rondônia State, in the western Amazon Basin of Brazil.

## Materials and methods

During entomological studies carried out between 2013 and 2015, in the municipalities of Alvorada d’Oeste, Buritis, Cacoal, Costa Marques, Espigão d’Oeste, Guajará-Mirim, Pimenta Bueno, Porto Velho and São Francisco Guaporé *Culicoides* specimens were collected with HP light traps in forests, pasture (livestock grazing) and peridomicilies (including animal shelters such as pigsties, henhouses and dog kennels).

*Culicoides* specimens were preserved in 70% ethanol, and then dissected and slide-mounted in phenol-balsam as described by Wirth and Marston (1968). The specimens were identified using the identification keys of Wirth and Blanton (1959), Spinelli et al. (1993), Felippe-Bauer et al. (2013). Subgeneric classification of *Culicoides* species was based on Borkent and Spinelli (2007) and Borkent (2016b). The specimens studied were deposited in the Ecology Laboratory of Infectious Disease in Amazon, Leonidas and Maria Deane Institute (ILMD), and the National Institute of Amazonian Research - Invertebrates Collection (INPA), Manaus, Amazonas, Brazil.

## Taxon treatments

### Culicoides (Hoffmania) heliconiae

Fox & Hoffman, 1944

Culicoides (Hoffmania) heliconiae Synonyms: *C. rozeboomi* Barbosa, 1947

#### Materials

- **Type status:** Other material. **Occurrence:** recordedBy: J.F. Medeiros; individualCount: 1; sex: male; lifeStage: adult; **Location:** country: Brazil; stateProvince: Rondônia; municipality: Cacoal; verbatimCoordinates: 11°25'53"S; 61°26'52"W11°25'53"S; 61°26'52"W; **Event:** samplingProtocol: HP light traps; eventDate: 2014-11-12/21; habitat: fragmented forest
- **Type status:** Other material. **Occurrence:** recordedBy: L.P.C. Carvalho; individualCount: 1; sex: female; lifeStage: adult; **Location:** country: Brazil; stateProvince: Rondônia; municipality: Buritis; verbatimCoordinates: 10°12'47"S; 63°53'46"W10°12'47"S; 63°53'46"W; **Event:** samplingProtocol: HP light traps; eventDate: 2015-05-26; habitat: forest

#### Diagnosis

Third palpal segment with scattered sensilla; apical pale band on mid femur, hind femur dark to tip; wing with distal pale spot in r_3_ which is narrow and transverse, r_3_ with pale spot present anterior to base of M_1_, a single pale spot crosses the second radial cell, apices of CuA_1_ and CuA_2_ pale; spermathecae with short, slender necks (Felippe-Bauer et al. 2009).

#### Distribution

Belize to Venezuela, Ecuador, Brazil (Acre, Amazonas, Pará and now in Rondônia), Grenada and Trinidad and Tobago (Borkent and Spinelli 2007, Farias et al. 2016a, Fox and Hoffman 1944 and Santarém and Felippe-Bauer 2017).

#### Notes

This species was here recorded for the first time in Rondônia State.

### Culicoides (Hoffmania) ignacioi

Forattini, 1957

Culicoides (Hoffmania) ignacioi Synonyms: *C. saintjusti* Tavares & Ruiz, 1980

#### Materials

- **Type status:** Other material. **Occurrence:** recordedBy: L.P.C. Carvalho; individualCount: 1; sex: male; lifeStage: adult; **Location:** country: Brazil; stateProvince: Rondônia; municipality: Porto Velho; locationRemarks: Pesque e Pague Farm; verbatimCoordinates: 08°40'02"S; 63°42'14"W08°40'02"S; 63°42'14"W; **Event:** samplingProtocol: HP light traps; eventDate: 2015-05-13/14; habitat: pasture
- **Type status:** Other material. **Occurrence:** recordedBy: J.F. Medeiros; individualCount: 3; sex: female; lifeStage: adult; **Location:** country: Brazil; stateProvince: Rondônia; municipality: Guajará-Mirim; verbatimCoordinates: 10°47'18"S; 65°20'32"W10°47'18"S; 65°20'32"W; **Event:** samplingProtocol: HP light traps; eventDate: 2013-10; habitat: forest

#### Diagnosis

Eyes contiguous, separated by a distance equal to the diameter of 1.5 ommatidial facets; third palpus segment broad in middle, with conspicuous irregular pit; wing as shown in figure; halter knob dark brown, pedicel pale. This species is similar to *C. fernandoi*, but can be distinguished by the mandible which has 20–22 teeth (14–15 in *C. fernandoi*), vein R_3_ pale (infuscated on lower portion with a very small dark spot behind apex in *C. fernandoi*), distal pale spot in cell r_3_ large, transverse (crescent-shaped or subdivided in *C. fernandoi*) (Spinelli et al. 1993).

#### Distribution

Brazil (Acre, Amazonas, Pará, Roraima, Maranhão, Minas Gerais, Rio de Janeiro, São Paulo and now in Rondônia) and Paraguay (Borkent and Spinelli 2007, Farias et al. 2016a, Forattini 1957 and Santarém and Felippe-Bauer 2017).

#### Notes

This species was here recorded for the first time in Rondônia State.

### Culicoides carpenteri

Wirth & Blanton, 1953

#### Materials

- **Type status:** Other material. **Occurrence:** recordedBy: J.F. Medeiros; individualCount: 1; sex: female; lifeStage: adult; **Location:** country: Brazil; stateProvince: Rondônia; municipality: Costa Marques; verbatimCoordinates: 12°24'25"S; 64°14'26"W12°24'25"S; 64°14'26"W; **Event:** samplingProtocol: HP light traps; eventDate: 2015-05-22; habitat: forest

#### Diagnosis

Third palpal segment scarcely swollen, with a broad, shallow sensory pit; second radial cell very dark, large pale area over r-m crossvein, extensive distal pale spot in cell r_3_ broadly extending across cell near apex from anterior wing margin to vein M_1_; apices of veins M_1_, M_2_ and CuA_1_ dark; subapical pale spot in cell m_2_, distal pale spot in cell m_1_ broadly meeting wing margin; two pale spots in distal part of cell m_2_, the distal one broadly meeting wing margin; the proximal one connected by a pale area extending to base of cell and including the pale spots lying in front of mediocubital fork and behind medial fork; pale area in cell CuA_1_ nearly filling entire cell; anal cell pale except for a large dark area centering on middle of stem of mediocubital vein; halter pale (Wirth and Blanton 1959).

#### Distribution

Costa Rica, Panama, Ecuador, Bolivia and Brazil (Amazonas and Rondônia) (Borkent and Spinelli 2007, Farias et al. 2016a, Santarém and Felippe-Bauer 2017 and Wirth and Blanton 1953).

#### Notes

This species was here recorded for the first time in Rondônia State.

### Culicoides dasyophrus

Macfie, 1940

#### Materials

- **Type status:** Other material. **Occurrence:** recordedBy: J.F. Medeiros; individualCount: 1; sex: female; lifeStage: adult; **Location:** country: Brazil; stateProvince: Rondônia; municipality: Cacoal; verbatimCoordinates: 11°25'53"S; 61°26'52"W11°25'53"S; 61°26'52"W; **Event:** samplingProtocol: HP light traps; eventDate: 2014-11-12/21; habitat: forest

#### Diagnosis

Eyes narrowly separated above, with long interfacetal hairs; third palpal segments swollen, with a broad, shallow, sensory pit; wing with poststigmatie pale spots in cell r_3_ more or less fused, the posterior one located slightly proximad of the anterior one, distal pale spot in cell r_3_ small, only one small pale spot in distal part of anal cell and one pale spot in distal part of cell m_2_; halter whitish; spermatheca one, pyriform (Wirth and Blanton 1956).

#### Distribution

Colombia, Venezuela, Ecuador, Guyana and Brazil (Amazonas, Pará, Roraima, Mato Grosso and now in Rondônia) (Borkent and Spinelli 2007, Farias et al. 2016a, Macfie 1940 and Santarém and Felippe-Bauer 2017).

#### Notes

This species was here recorded for the first time in Rondônia State.

### Culicoides eublepharus

Macfie, 1948

Culicoides eublepharus Synonyms: *C. transferrans* Ortiz, 1953

#### Materials

- **Type status:** Other material. **Occurrence:** recordedBy: J.F. Medeiros; individualCount: 1; sex: female; lifeStage: adult; **Location:** country: Brazil; stateProvince: Rondônia; municipality: Cacoal; verbatimCoordinates: 11°25'53"S; 61°26'52"W11°25'53"S; 61°26'52"W; **Event:** samplingProtocol: HP light traps; eventDate: 2014-11-12/21; habitat: forest fragment

#### Diagnosis

Eyes narrowly separated, with long interfacetal hairs; third palpal segment moderately swollen, with a broad, shallow, sensory pit; wing with four pale spots in a rhomboid in cell r_3_, the distal pair usually fused and broadly attaining wing margin anteriorly; two pale spots each in cells m_1_ and apices of cells m_2_ and anal cell; pale spots present behind medial fork and in front of mediocubital fork; halter yellowish; spermatheca one, oval with a short portion of the duct sclerotized (Wirth and Blanton 1959).

#### Distribution

Mexico, Costa Rica to Venezuela and Ecuador and Brazil (Amazonas, Pará, Roraima and now in Rondônia) (Borkent and Spinelli 2007, Farias et al. 2016a, Macfie 1948 and Santarém and Felippe-Bauer 2017).

#### Notes

This species was here recorded for the first time in Rondônia State.

### Culicoides galindoi

Wirth & Blanton, 1953

#### Materials

- **Type status:** Other material. **Occurrence:** recordedBy: J.F. Medeiros; sex: 1 female, 1 male; lifeStage: adult; **Location:** country: Brazil; stateProvince: Rondônia; municipality: Cacoal; verbatimLocality: Chácara do Gerson and Rancho Cassiano; verbatimCoordinates: 11°25'53"S; 61°26'52"W11°25'53"S; 61°26'52"W; **Event:** samplingProtocol: HP light traps; eventDate: 2014-11-12/21; habitat: forest fragment

#### Diagnosis

Eyes contiguous, bare; third segment slightly swollen, with a small, shallow, sensory pit; pale distal spot in cell r_3_ rounded distally, leaving a small dark area in apex of cell; halter pale; spermathecae two, pyriform, subequal (Wirth and Blanton 1959).

#### Distribution

Costa Rica, Panama, and Brazil (Acre, Amazonas, Maranhão, Roraima and now in Rondônia) (Borkent and Spinelli 2007, Farias et al. 2016a, Santarém and Felippe-Bauer 2017 and Wirth and Blanton 1953).

#### Notes

This species was here recorded for the first time in Rondônia State.

### Culicoides (Haematomyidium) darlingtonae

Wirth & Blanton, 1971

#### Materials

- **Type status:** Other material. **Occurrence:** recordedBy: J.F. Medeiros; sex: 2 females, 3 males; lifeStage: adult; **Location:** country: Brazil; stateProvince: Rondônia; municipality: Espigão do Oeste; verbatimCoordinates: 11°67'63"S; 60°69'90"W11°67'63"S; 60°69'90"W; **Event:** samplingProtocol: HP light traps; eventDate: 2014-04; habitat: forest
- **Type status:** Other material. **Occurrence:** recordedBy: J.F. Medeiros; sex: 3 females, 5 males; lifeStage: adult; **Location:** country: Brazil; stateProvince: Rondônia; municipality: Pimenta Bueno; verbatimLocality: Usina Hidrelétrica Rondon II; verbatimCoordinates: 11°96'48"S; 60°69'90"W11°96'48"S; 60°69'90"W; **Event:** samplingProtocol: HP light traps; eventDate: 2014-10-10/11; habitat: fragment forest
- **Type status:** Other material. **Occurrence:** recordedBy: J.F. Medeiros; individualCount: 1; sex: female; lifeStage: adult; **Location:** country: Brazil; stateProvince: Rondônia; municipality: Cacoal; verbatimLocality: Rancho Cassiano; verbatimCoordinates: 11°25'53"S; 61°26'52"W11°25'53"S; 61°26'52"W; **Event:** samplingProtocol: HP light traps; eventDate: 2014-11-12/21; habitat: fragment forest

#### Diagnosis

Eyes narrowly separated, with short interfacetal hairs; third palpal segment considerably swollen its full length, with shallow and round sensory pit; proboscis short; thorax dark brown; legs brown, knee spots blackish; second radial cell included in a dark spot to its apex, cell r_3_ with contiguous poststigmatic pale spots, cell m_1_ with two pale spots, cell m_2_ with small pale spot; two spermathecae and rudimentary third, with sclerotized ring (Wirth and Blanton 1971).

#### Distribution

Costa Rica, Trinidad and Brazil (Rondônia) (Borkent and Spinelli 2007, Farias et al. 2016a, Santarém and Felippe-Bauer 2017 and Wirth and Blanton 1971).

#### Notes

This species was here recorded for the first time in Brazil.

## Discussion

The major *Culicoides* species collected in this study are distributed throughout the Brazilian Amazon (Farias et al. 2016a, Santarém and Felippe-Bauer 2017); *C. dasyophrus*, *C. eublepharus*, *C. galindoi* and *C. ignacioi* are found from Acre State to Maranhão State (Santarém and Felippe-Bauer 2017). The specimens of *C. heliconiae* herein were collected in a forest environment, which is the same habitat as the specimens collected by Fittkau in 1970 in APEG Forest, Pará State (Wirth and Blanton 1973). The main breeding sites this species are water-holding plants, rotting parts or fruits of plants (Wirth and Blanton 1968).

*Culicoides ignacioi* is distributed throughout Paraguay and Brazil (Borkent and Spinelli 2007). In Brazil, this species has been recorded in the states of Acre, Amazonas, Pará, Roraima, Maranhão, Minas Gerais, Rio de Janeiro and São Paulo (Santarém and Felippe-Bauer 2017). The specimens herein were collected in a forest environment. Silva and Carvalho (2013), sampled *Culicoides* from many points of collection and this species was captured in highest abundance in Cerrado forest, in the State of Maranhão.

*Culicoides eublepharus* has been recorded in Mexico, Costa Rica, Venezuela, Ecuador and Brazil. It has been found in the Brazilian Amazon in states of Amazonas, Pará and Roraima (Borkent and Spinelli 2007, Farias et al. 2016a). The specimens herein were collected in forest fragments. Veras and Castellon (1998) collected this species in forests on the Adolpho Ducke Forest Reserve, in Amazonas State.

This study increases the number of species recorded in Rondônia State to 40. Of the seven species here recorded, all are new records in Rondônia state, and one is also a new record in Brazil; underlining how poorly the distribution of even medically important insects such as *Culicoides* still is understood in Brazil. This study shows that species diversity is high among Amazonian *Culicoides*. Furthermore, this study will be helpful in knowledge of *Culicoides* fauna in the Amazon, and may contribute to a better understanding of the medical importance and vector epidemiology these insects.

## Supplementary Material

XML Treatment for Culicoides (Hoffmania) heliconiae

XML Treatment for Culicoides (Hoffmania) ignacioi

XML Treatment for Culicoides carpenteri

XML Treatment for Culicoides dasyophrus

XML Treatment for Culicoides eublepharus

XML Treatment for Culicoides galindoi

XML Treatment for Culicoides (Haematomyidium) darlingtonae
